# Maximizing Negative Correlations in Resting-State Functional Connectivity MRI by Time-Lag

**DOI:** 10.1371/journal.pone.0111554

**Published:** 2014-11-14

**Authors:** Gadi Goelman, Noam Gordon, Omer Bonne

**Affiliations:** 1 MRI/MRS Lab, The Human Biology Research Center, Department of Medical Biophysics, Hadassah Hebrew University Medical Center, Jerusalem, Israel; 2 Department of Psychiatry, Hadassah Hebrew University Medical Center, Jerusalem, Israel; Italian Institute of Technology, Italy

## Abstract

This paper aims to better understand the physiological meaning of negative correlations in resting state functional connectivity MRI (*r-fcMRI*). The correlations between anatomy-based brain regions of 18 healthy humans were calculated and analyzed with and without a correction for global signal and with and without spatial smoothing. In addition, correlations between anatomy-based brain regions of 18 naïve anesthetized rats were calculated and compared to the human data. T-statistics were used to differentiate between positive and negative connections. The application of spatial smoothing and global signal correction increased the number of significant positive connections but their effect on negative connections was complex. Positive connections were mainly observed between cortical structures while most negative connections were observed between cortical and non-cortical structures with almost no negative connections between non-cortical structures. In both human and rats, negative connections were never observed between bilateral homologous regions. The main difference between positive and negative connections in both the human and rat data was that positive connections became less significant with time-lags, while negative connections became more significant with time-lag. This effect was evident in all four types of analyses (with and without global signal correction and spatial smoothing) but was most significant in the analysis with no correction for the global signal. We hypothesize that the valence *of r-fcMRI* connectivity reflects the relative contributions of cerebral blood volume (CBV) and flow (CBF) to the BOLD signal and that these relative contributions are location-specific. If cerebral circulation is primarily regulated by CBF in one region and by CBV in another, a functional connection between these regions can manifest as an *r-fcMRI* negative and time-delayed correlation. Similarly, negative correlations could result from spatially inhomogeneous responses of rCBV or rCBF alone. Consequently, neuronal regulation of brain circulation may be deduced from the valence of *r-fcMRI* connectivity.

## Introduction

Coherent low frequency fluctuations of the blood oxygenation level-dependent (BOLD) signal in the resting-state were shown to contain functional neuronal network information [1], [2]. Such information is derived from correlations between the temporal fluctuations of the BOLD signal in various brain regions in the absence of external stimuli [3]–[5]. Multiple resting-state networks (RSN) were defined [3], [6], [7] in this manner, and their reliability and robustness were established [8]–[10]. RSNs were also shown in anesthetized animals [11], [12]. Most RSN sets show positive correlations between the brain regions comprising these networks. However, several RSNs were shown to be inversely correlated. For example, it was shown that the default mode network is negatively correlated with the dorsal attention system [4], [5]. Alterations in such anti-correlated networks between healthy subjects and patients with, for example, schizophrenia [13], ADHD [14], bipolar disorder [15] and Alzheimer's disease [16] were observed.

The physiological mechanisms underlying resting-state functional brain connectivity MRI (r-fcMRI) are however, not clear. Positive correlations between regions comprising such networks are assumed to reflect synchronized activity between these regions, but the nature of negative correlations is debatable. A distinction should be made between *physiological* sources of negative correlations, such as negative BOLD signals [17]–[26], and possible data analysis biases [27]–[31]. Several studies have demonstrated negative correlations using methodologies that are free of such biases [32], [33], thereby strengthening the assumption that both negative and positive correlations reflect genuine physiological processes. Potential physiological sources for negative correlations within resting state networks are neuronal inhibition [26], [34] and pure non-neuronal hemodynamic processes [31].

In this study we aim to better understand the physiological mechanisms underlying negative correlations in *r-fcMRI*. Since the distinction between positive and negative correlations may be made difficult by the presence of a global effect that biases all correlations toward positive values (for example see [30]), we analyzed our *r-fcMRI* data with and without correction for the global signal. We also tested the effect of spatial smoothing by analyzing the data with and without smoothing. We then compared human and rat *r-fcMRI* data, trying to account for both similarities (brain organization and functionality) and differences (hemodynamic functions) in cerebral function among the species. Based on our findings we conclude that negative and positive correlations have distinct physiological properties and propose a mechanism for negative correlations in r-fcMRI that accounts for all our findings.

## Methods

### Human data

Eighteen human data-sets of healthy subjects (age 29.2±7.4; 8 males and 10 females) were downloaded from the NITRC site (http://www.nitrc.org/projects/fcon_1000/). Data was generated by professors Milham, M.P. and Castellanos, F.X. groups' and generously posted in this site for public use (data taken from NewYork_a_part1 and part2). Data sets were chosen randomly without any exclusion criteria. To allow reproduction of the results, analysis was performed in the ‘Data Processing Assistant for Resting-State fMRI (DPARSF) Advanced Edition' (Release  = V2.3_130615, http://www.restfmri.net) [35] which is based on Statistical Parametric Mapping (SPM8, Welcome Department, London UK) and Resting-State fMRI Data Analysis Toolkit [36], thus available to the public. Images were realigned, co-registered to T1 anatomy, segmented, normalized, either smoothed by a [4×4×4] voxel kernel or not smoothed, de-trended, filtered (0.01<>0.08 Hz), covaried by the 6 rigid body functions and either with or without the global signal and then scrubbed (FD>0.5 with ‘bad’ data points removed [37]). Using a WFU PickAtlas toolbox [38], [39], 57 ROIs (36 cortical and 21 non-cortical) were selected in the MNI space (Table 1) covering the extended limbic system. ROIs were implemented in the DPARSFA toolbox, functional connectivity between them was calculated and their time courses were extracted. The analyses yielded four connectivity analysis sets. (1) Without spatial smoothing and without global regression (marked hereafter as ‘-S –G’). (2) With spatial smoothing and without global regression (‘+S –G’). (3) Without smoothing but with global regression (‘-S +G’) and (4) with smoothing and with regression (‘+S +G’). All further analysis was performed in custom-made IDL software.

**Table 1:** Regions of interest (ROIs) in the human brain, their mean MNI coordinates and number of voxels.

*#*	*Name*	*X*	*Y*	*Z*	*Volume*
0	R amygdala	25.1	−0.2	−18.5	34
1	L amygdala	−21.4	−0.3	−18.8	30
2	R dmPFC	19.9	37.3	34.3	115
3	L dmPFC	−22.6	37.2	32.9	119
4	R dorsolateral PFC	34.1	50.1	29.4	27
5	L dorsolateral PFC	−30.8	49.6	29.3	30
6	R dmPFC -b	19.5	61.3	24.8	41
7	L dmPFC -b	−16.4	61.3	24.6	41
8	R habenula	7.6	−20.0	4.9	43
9	L habenula	−4.5	−20.2	4.8	43
10	R medial BA8-b	23.6	17.4	50.8	415
11	L medial BA8-b	−20.3	17.6	50.8	419
12	R medial BA8	13.6	39.5	48.9	208
13	L medial BA8	−10.4	39.5	48.7	215
14	R Caudate	12.4	10.8	9.8	137
15	L Caudate	−9.4	10.4	9.7	140
16	R medial BA9	37.3	21.4	36.6	335
17	L medial BA9	−34.6	21.6	36.7	328
18	R GP	20.6	−1.4	−0.6	75
19	L GP	−16.1	−1.0	−0.3	72
20	R hippocampus	31.6	−17.1	−14.3	23
21	L hippocampus	−27.7	−17.9	−14.1	29
22	R medial PFC	5.5	67.6	−3.3	56
23	L medial PFC	−2.2	67.8	−3.0	62
24	R NAc	11.6	13.3	−7.7	121
25	L NAc	−8.8	13.2	−7.8	122
26	R posterior Cingulate-b	9.4	−48.5	14.6	416
27	L posterior Cingulate-b	−6.5	−48.7	14.7	423
28	R pregenual ACC	11.6	31.5	22.7	85
29	L pregenual ACC	−8.6	31.5	22.7	86
30	R insula	41.2	−3.9	10.1	492
31	L insula	−36.9	−3.6	10.1	494
32	R mammillary	9.0	−12.5	−4.4	2
33	L mammillary	−6.5	−14.5	−1.9	3
34	R STN	11.3	−11.0	−5.3	4
35	L STN	−8.3	−11.0	−5.3	4
36	R subgenual ACC	7.6	33.4	0.5	281
37	L subgenual ACC	−4.5	33.6	0.5	279
38	R subgenual cingulate	3.2	27.7	−19.3	124
39	L subgenual cingulate	−0.2	27.5	−19.3	123
40	R ventral striatum	21.9	15.3	−1.3	43
41	L ventral striatum	−14.6	11.4	−5.4	42
42	R ParaHippocampal	26.7	−13.0	−21.1	246
43	L ParaHippocampal	−19.9	−13.5	−21.2	231
44	R Posterior Cingulate	7.4	−40.7	40.7	281
45	L Posterior Cingulate	−4.4	−40.5	40.6	283
46	R putamen	25.5	3.5	3.0	198
47	L putamen	−21.6	3.2	3.1	196
48	R SN	12.0	−13.5	−11.3	6
49	L SN	−7.5	−13.3	−11.9	4
50	R visual BA19	5.7	−72.4	30.3	109
51	L visual BA19	−2.6	−72.9	30.7	111
52	R supp motor	9.9	2.2	61.2	541
53	L supp motor	−4.0	6.6	60.6	523
54	Medial BA9	3.7	51.5	22.5	37
55	Superior precuneus	3.3	−56.6	38.6	320
56	DRN	1.5	29.4	−17.3	340

Abbreviations: dmPFC, dorsal medial prefrontal cortex; PFC, prefrontal cortex; BA, Brodman area; GP, globus pallidus; NAc, nucleus accumbens; M1, primary motor; M2, secondary motor; SIJ, primary sensory cortex (jaw region); CPu, caudate putamen; GP, globus pallidus; STN, subthalamic nucleus; ACC, anterior cingulate cortex; DRN, dorsal raphe nucleus.

### Rat data

The study was approved by the *Animal Care and Use Committee of the Hebrew University*. Experiments were carried out in accordance with the NIH Guidelines regarding the care and use of animals for experimental procedures (NIH approval number: OPRR-A01-5011).18 male Sprague-Dawley (380–450 g, age 19±2 weeks supplied by Harlan, Rehovot Israel) rats were included in the study.

Data were collected with a 4.7T Bruker BioSpec scanner (Bruker Biospin Ettlington, Germany) using a Dotty quadrature rat head coil. Rats were anesthetized with isoflurane (1.5% +30∶70 O_2_∶N_2_O). Respiration rates were continuously monitored and were held between 55–65 min^−1^ by small adjustments of the isoflurane concentration. Body temperature was kept stable (37°C ±1) using a water heating bed. 2D T2-weighted coronal images were acquired for anatomy. Functional BOLD contrast MRI was collected with EPI-FID (TR = 2, TE = 20 ms, 300 repetitions, matrix = 128×64×15, FOV  = 3×3 cm^2^, 1mm slice width and three sequential sets). Functional data was first processed in SPM8 using standard spatial preprocessing steps. Images were slice-time corrected, realigned and resliced. At a second step, analysis was performed using custom-made IDL and Matlab software. It included regression-out the six functions related to motion, alignment of the MRI images to the rat brain atlas[40], data smoothing by a 3-point-Gaussian kernel, and band-pass filtering (0.01<>0.1 Hz). 69 ROIs (24 cortical and 45 non-cortical) were pre-selected in the atlas (Table 2) covering the extended limbic system. Correlations between predefined ROIs were obtained by calculating Pearson correlations between all possible pairs and applying Fischer's z transformation.

**Table 2:** Regions of interest (ROIs) in the rat brain, their mean coordinates and volume in ml.

*#*	*Name*	*X*	*Y*	*Z*	*Volume*
0	R M1 (rostral)	3.4	2.6	3.7	7.00
1	L M1 (rostral)	−3.5	2.6	3.7	6.96
2	R M2 (rostral)	1.3	1.6	3.7	2.39
3	L M2 (rostral)	−1.4	1.6	3.7	2.38
4	R cingulate area 1	0.6	2.1	1.9	5.36
5	L cingulate area 1	−0.7	2.1	1.9	5.28
6	R pre-limbic	0.6	3.7	3.3	4.07
7	L pre-limbic	−0.7	3.7	3.3	4.10
8	R infra-limbic	0.4	5.0	3.2	1.20
9	L infra-limbic	−0.5	5.0	3.1	1.23
10	R frontal area 3	4.2	4.2	3.7	1.34
11	L frontal area 3	−4.2	4.2	3.7	1.35
12	R insular	3.8	5.1	3.7	2.62
13	L insular	−3.9	5.1	3.7	2.62
14	R NAc core	1.5	6.6	2.7	1.27
15	L NAc core	−1.5	6.6	2.7	1.29
16	R NAc shell	1.2	7.4	2.7	1.02
17	L NAc shell	−1.2	7.4	2.7	1.06
18	R sensory SIJ	5.0	4.5	2.3	8.57
19	L sensory SIJ	−5.0	4.5	2.3	8.71
20	R CPu rostral	2.8	5.5	1.2	17.92
21	L CPu rostral	−2.9	5.5	1.2	18.00
22	R cingulate area 2	0.5	3.1	0.9	3.73
23	L cingulate area 2	−0.6	3.1	0.9	3.60
24	R CPU caudal	3.8	5.8	−0.6	12.77
25	L CPU caudal	−3.8	5.7	−0.6	13.14
26	R basal nucleus	3.2	8.0	−1.3	0.15
27	L basal nucleus	−3.2	7.9	−1.3	0.18
28	R GP,	3.5	7.0	−1.6	2.80
29	L GP	−3.6	6.9	−1.6	2.91
30	R VPL	2.8	6.4	−2.3	1.35
31	L VPL	−3.0	6.4	−2.3	1.21
32	R VA+VL	1.9	6.1	−2.3	2.20
33	L VA+VL	−2.0	6.2	−2.3	2.20
34	R EP	2.8	7.9	−2.3	0.33
35	L EP	−2.9	7.9	−2.3	0.22
36	R S1BF	5.3	3.0	−2.3	5.77
37	L S1BF	−5.4	3.0	−2.3	5.76
38	R amygdala	4.2	9.2	−2.6	9.88
39	L amygdala	−4.4	9.2	−2.5	8.78
40	R Hab	0.7	5.0	−3.3	0.48
41	L Hab	−0.7	5.0	−3.3	0.48
42	R Po (thalamus)	2.2	5.9	−3.3	2.03
43	L Po (thalamus)	−2.3	5.8	−3.3	1.84
44	R hippocampus	2.2	3.3	−3.3	5.55
45	L hippocampus	−2.5	3.3	−3.3	6.31
46	R granular cortex	0.6	1.7	−4.7	7.47
47	R granular cortex	−0.7	1.7	−4.8	7.89
48	R hypothalamus	0.9	9.0	−3.3	2.96
49	L hypothalamus	−0.9	9.0	−3.3	2.64
50	R zona inserta	2.6	7.5	−4.3	0.99
51	L zona inserta	−2.8	7.5	−4.3	0.95
52	Mammillary	−0.1	9.3	−4.3	2.70
53	R SNr	2.3	8.4	−5.3	1.52
54	L SNr	−2.4	8.4	−5.3	1.40
55	R Red nucleus	1.0	7.5	−5.3	0.52
56	L Red nucleus	−1.1	7.5	−5.3	0.50
57	R VTA	0.6	8.2	−6.3	0.58
58	L VTA	−0.8	8.1	−6.3	0.53
59	R sup colliculus	1.3	4.1	−6.8	9.49
60	L sup colliculus	−1.4	4.2	−6.8	9.67
61	R DG	4.2	5.8	−6.3	5.89
62	L DG	−4.3	5.9	−6.3	6.03
63	IP	−0.1	9.0	−6.3	0.84
64	R pontine	0.8	10.1	−7.3	1.33
65	L pontine nuc	−0.9	10.1	−7.3	1.36
66	DRN	0.0	6.4	−7.3	1.16
67	R STN	2.6	8.1	−3.3	0.30
68	L STN	−2.7	8.1	−3.3	0.30

Abbreviations: M1, primary motor cortex; M2, secondary motor cortex; NAC, nucleus accumbens; SIJ, primary sensory cortex (jaw region); CPu, caudate-putamen; GP, globus pallidus; VPL, ventral posterolateral thalamic nucleus; VA, ventral anterior thalamic nucleus; VL, ventrolateral thalamic nucleus; EP, entopeduncular nucleus; S1BF, primary sensory cortex (barrel field); Hab, habenula; Po, post thalamic nucleus; SNr, substantia nigra; VTA, ventral tegmental area; DG, dentate gyrus; IP, interpeduncular nucleus; DRN, dorsal raphe nucleus; STN, subthalamic nucleus.

### Statistics

A one group t-statistic random effect analysis on the Fisher transformed values between subjects was performed. To correct for multiple comparisons, False Discovery Rate (FDR) correction with a threshold of α = 0.001 for humans and α = 0.01 for rats was used (the difference was due to the weaker effect in rats that is expected due to the anesthesia). To define significant connections, we used the lowest threshold amongst the four analyses (the threshold corresponding to ‘+S –G’). Each of the four analyses (-S-G; +S-G; -S+G; +S+G) had a FDR cutoff corresponding to α = 0.001. To minimize differences due to thresholds, the same cutoff was used in all groups. Connections whose p-values were lower than the threshold are termed hereafter 'significant connections'. Categorizing connections as 'positive' or 'negative' was done based on their t-values. Significant connections with positive t-values were named 'positive-connections' and significant connections with negative t-values were named 'negative connections'. For the definition of the t-values we subtracted the global mean correlation (all Fisher transformed values from the entire group) from all correlations and then divided it by the standard error (similarly to [41]). This resulted in distributions that were centered at zero.

### Effect of time lags

To test the effect of time-lags on the correlations, we calculated the correlation values for each significant connection introducing a time-lag ranging from −26 to 26 seconds. This time range was chosen based on reported BOLD signal post-stimulus length in humans [42] and in rats [43]. Specifically, we counted the number of positive connections whose correlations became more positive and the number of negative connections whose correlations became more negative as a function of the time-lag values.

## Results

**Figure 1:**
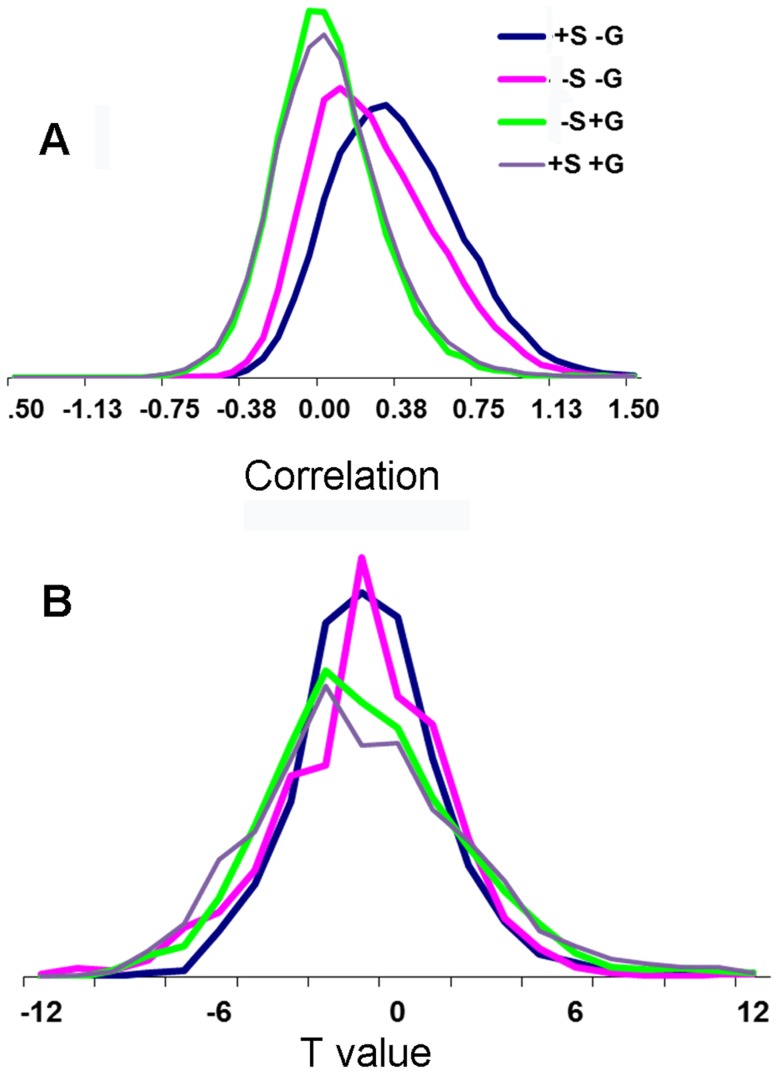
Distributions of Fisher transformed and of t-values for the four different analyses of the human data. *(A)* Distributions of Fisher transformed values. *(B)* Distributions of t-values. The analyses differed in either applying or not applying spatial smoothing and correction for global signal. ‘–S -G’ – analysis without smoothing and without global regression, ‘+S -G’ – analysis with smoothing and without global regression, ‘–S +G’ – analysis without smoothing and with global regression and ‘–S -G’ – analysis with smoothing and with global regression.

Figure 1 presents the distributions of the Fisher-transformed values and of the group t-values for all ROI pairs for the four different analyses of the human data. As expected [30], the distribution of the Fisher-transformed values of the uncorrected data centered on positive values and was shifted approximately to zero by removing the global signal. The distributions of t-values were, as expected, centered on zero but had different widths. The distribution of the Fisher-transformed values of the rat data was centered approximately on zero. For that reason no global signal removal was applied on the rat data.

**Figure 2:**
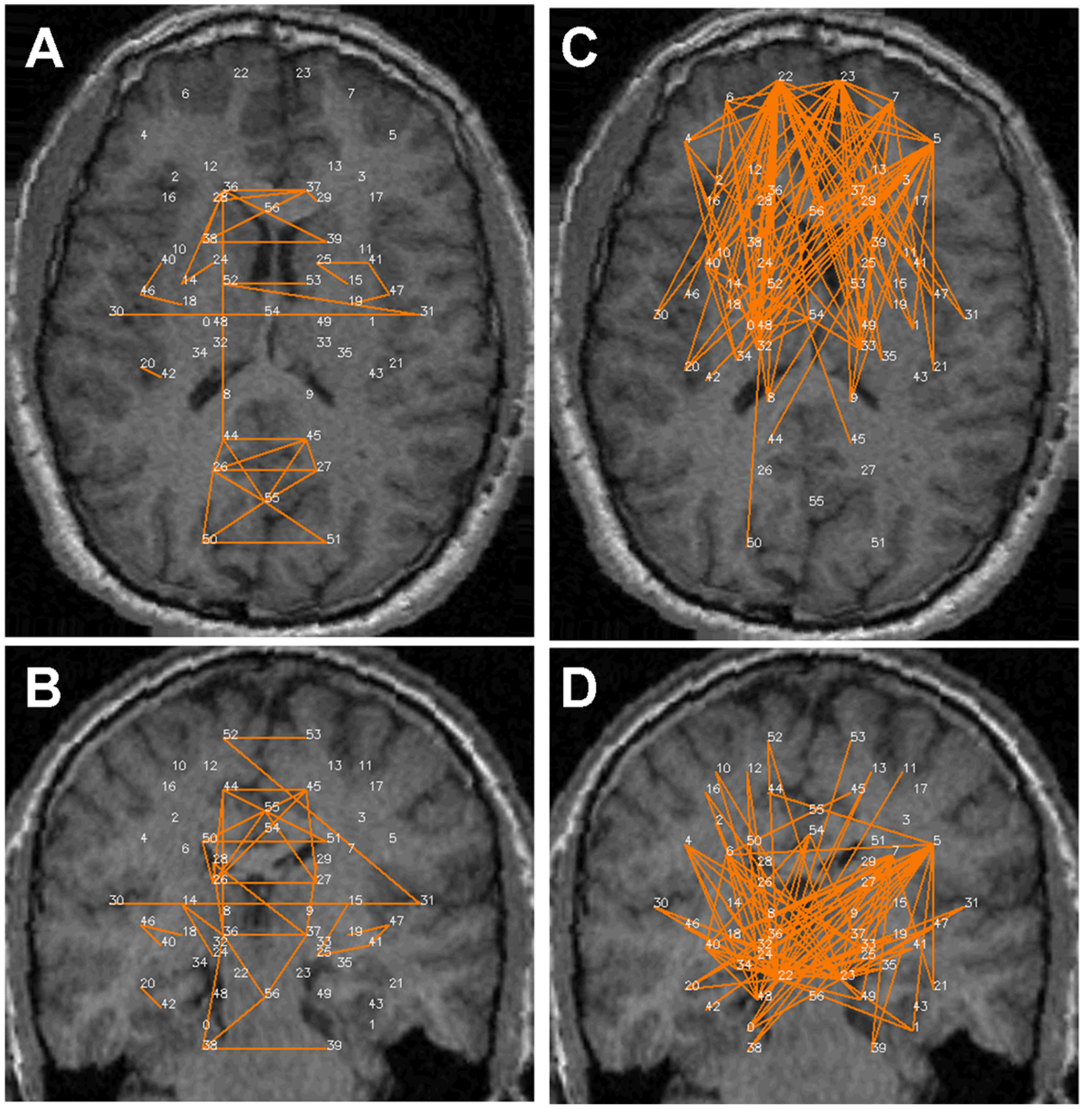
Significant connections within the 57 predefined human regions for the –S-G analysis. Significant connections are presented as 2D projections on top of T1-weighted coronal and axial MRI images. ROIs are annotated using numbers provided in Table 1. *A & B*. Positive connections. *C & D*. Negative connections.

**Figure 3:**
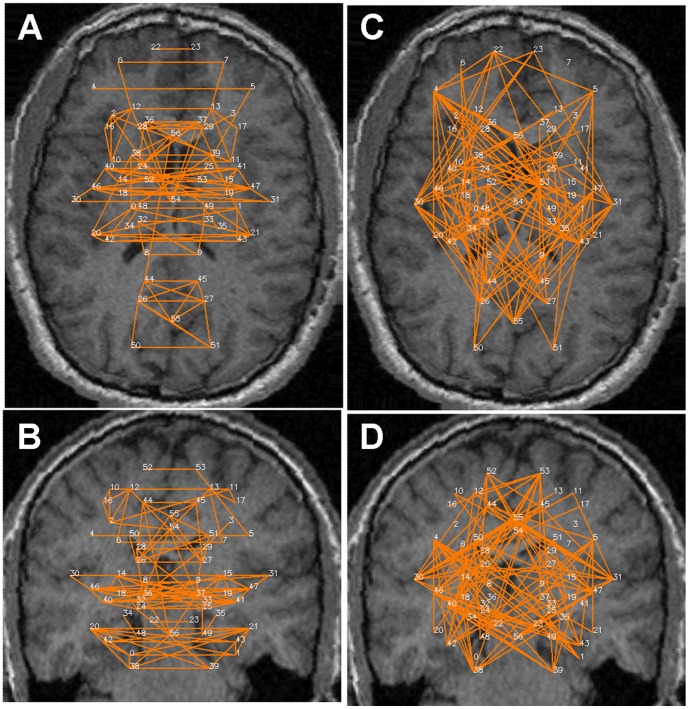
Significant connections within the 57 predefined human regions for the +S+G analysis. Significant connections are presented as 2D projections on top of T1-weighted coronal and axial MRI images. ROIs are annotated using numbers provided in Table 1. *A & B*. Positive connections. *C & D*. Negative connections.

**Figure 4:**
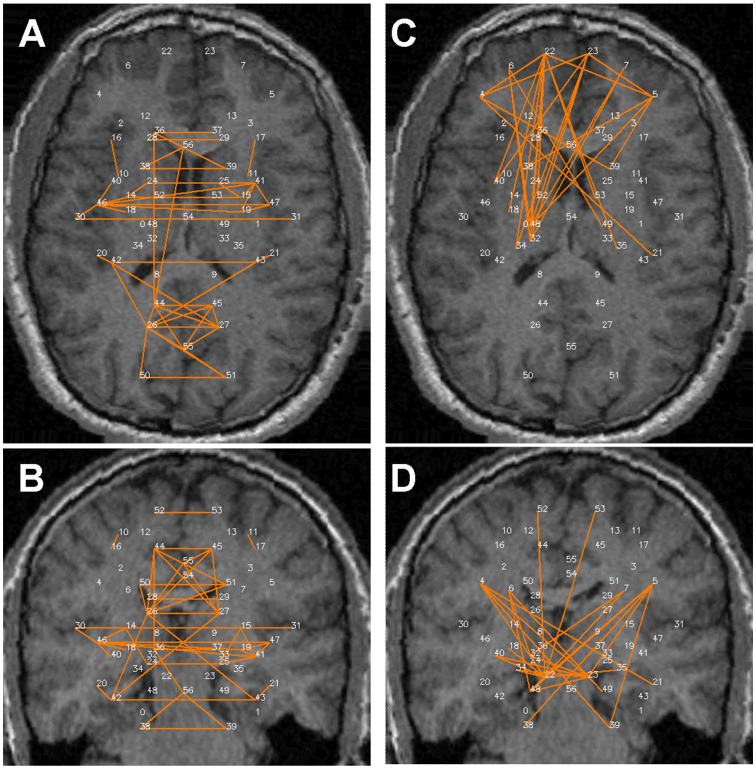
Significant connections within the 57 predefined human regions for the +S-G analysis. Significant connections are presented as 2D projections on top of T1-weighted coronal and axial MRI images. ROIs are annotated using numbers provided in Table 1. *A & B*. Positive connections. *C & D*. Negative connections.

**Figure 5:**
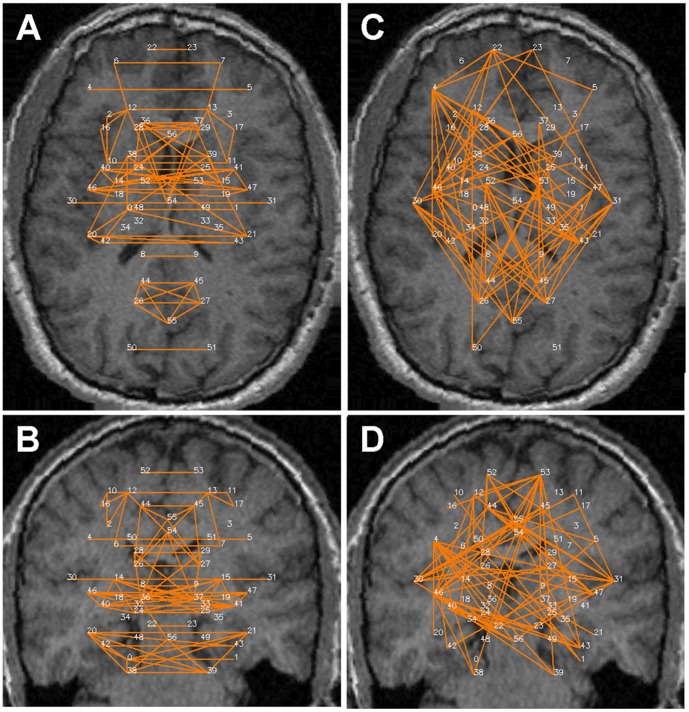
Significant connections within the 57 predefined human regions for the -S+G analysis. Significant connections are presented as 2D projections on top of T1-weighted coronal and axial MRI images. ROIs are annotated using numbers provided in Table 1. *A & B*. Positive connections. *C & D*. Negative connections.

**Figure 6:**
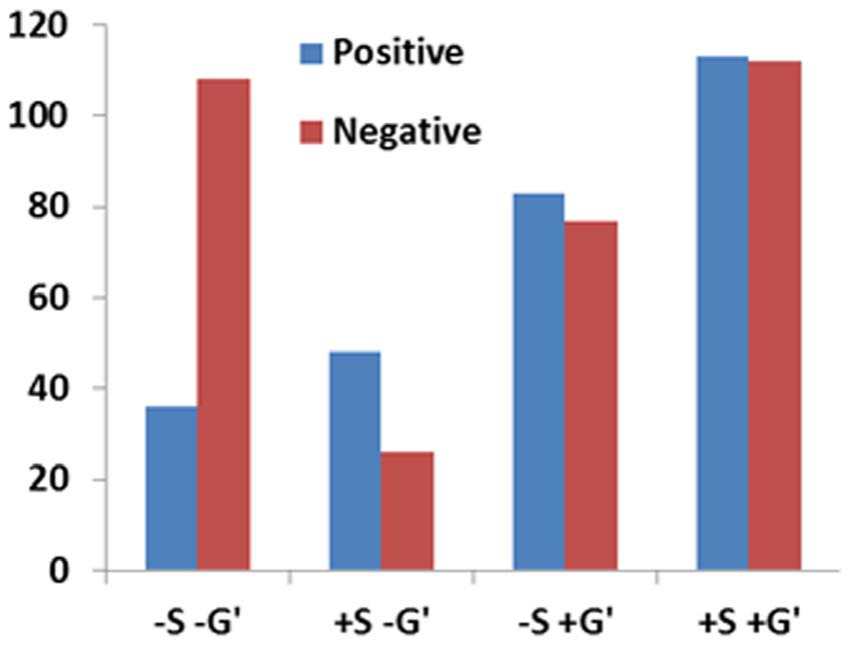
Number of significant positive and negative connections for the different human analyses.

Figures 2–5 show the significant positive (A & B) and significant negative (C & D) connections in the human data, each for a different analysis. In figure 6 we show the numbers of significant positive and significant negative connections that were obtained by these analyses in the human data. Whereas spatial smoothing and correction for the global signal increased the number of positive connections, the effect on the negative connections was more complex. To better quantify the differences between the analyses, we calculated the percentage of common connections (positive and negative separately) between the analyses. Table 3 shows that whereas most of the positive connections were common regardless of the analysis used, different negative connections were obtained when a correction for global signal was applied.

**Table 3:** Percentage of overlap between the significant connections obtained by the four types of analyses of the human data.

	-S-G	+S-G	-S+G	+S+G
**-S-G**		78(+)	75(+)	89(+)
**+S-G**	81(−)		78(+)	86(+)
**-S+G**	30(−)	14(−)		97(+)
**+S+G**	42(−)	16(−)	88(−)	

**Figure 7:**
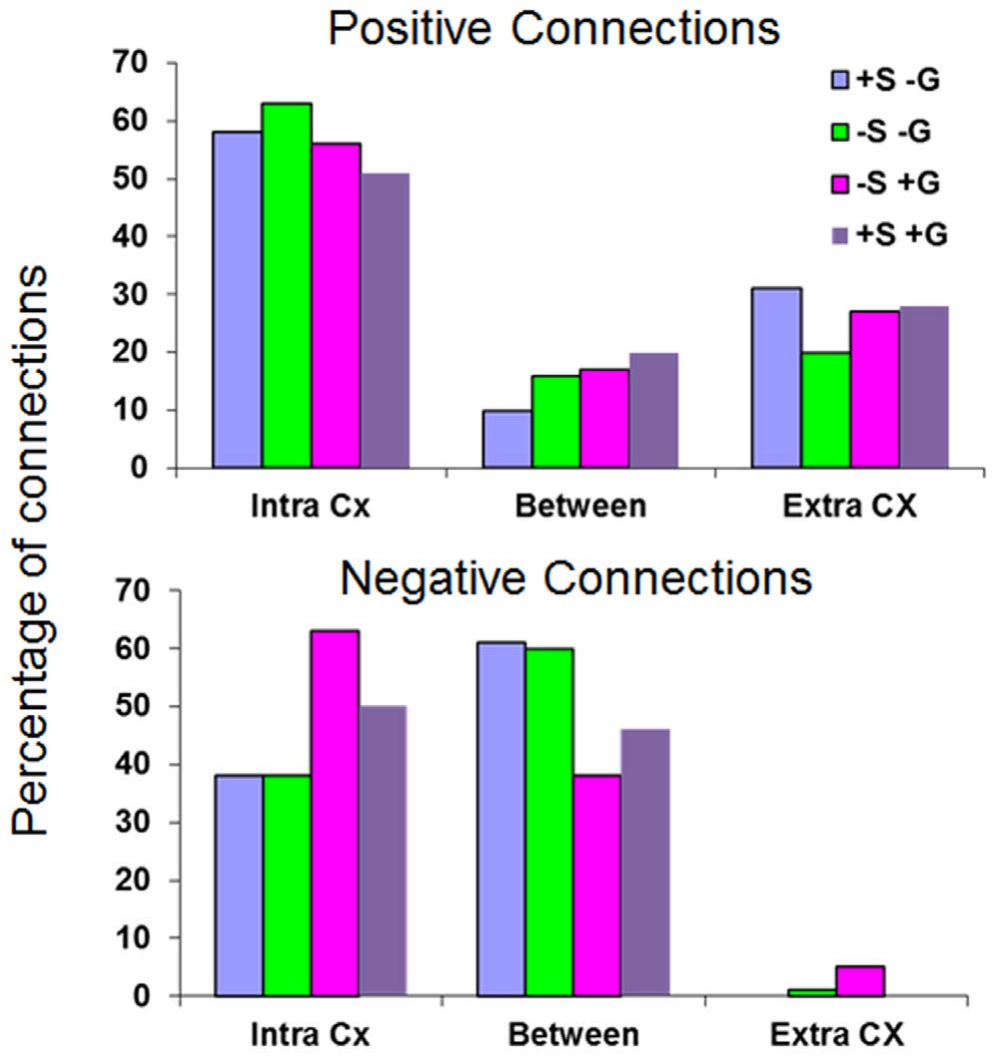
Categorization of positive and negative human connections. Percentages of significant connections into the ‘Intra Cx’ (between two cortical ROIs), the ‘Extra-Cx’ (between two non-cortical ROIs) and the ‘Between’ (between cortical and non-cortical ROIs) categories. *Top*. Positive connections. *Bottom*. Negative connections.

Percentage of overlap between positive connections is shown by (+) and between negative connections by (−). –S-G: analysis without spatial smoothing and without global signal regression; +S-G: analysis with spatial smoothing and without global signal regression; -S+G: analysis without spatial smoothing and with global signal regression; +S+G: analysis with spatial smoothing and with global signal regression;

Careful inspection of Figures 2–5 suggests that negative and positive connections differ with respect to the structures they connect. To quantify this point, we categorized all negative and positive significant connections into three types: those connecting between two cortical ROIs ("Intra-Cx"), those connecting between two non-cortical ROIs ("Extra-Cx"), and those connecting a cortical with a non-cortical ROI ("Between"). The percentages of these categories are shown in Figure 7 for the four different analyses of the human data. There was a clear distinction between positive and negative connections with almost no negative connections linking non-cortical regions and only few positive connections linking cortical and non-cortical regions. As seen in Figure 7, the effect of the different analyses on the distribution of positive connections was small while it was greater on the negative connections. Spatial smoothing had a relatively minor effect compared to the significant effect of global regression.

**Figure 8:**
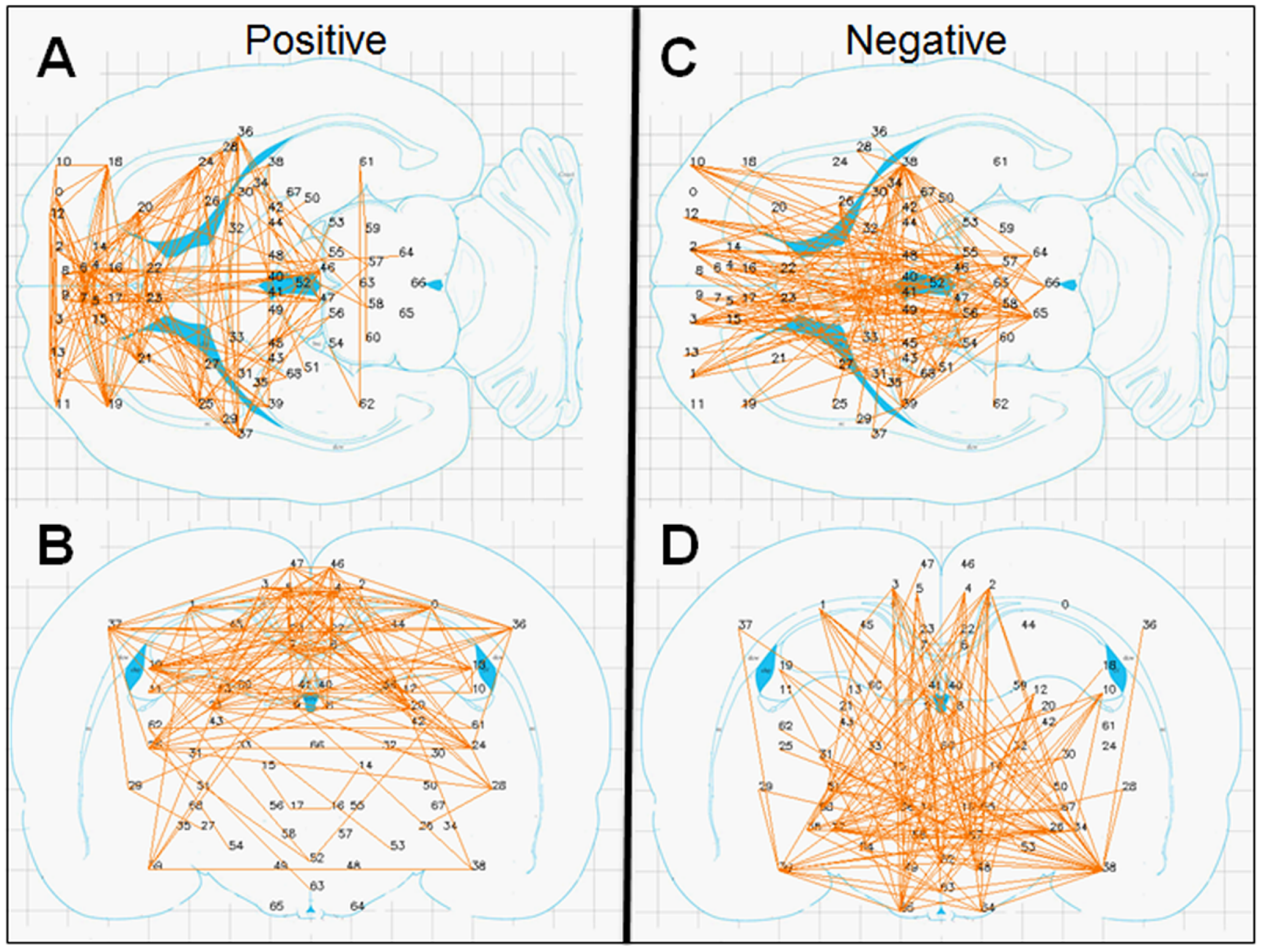
Significant connections within the 69 predefined rat regions. Significant connections are presented as 2D projections on top of coronal and axial Figures from the rat brain atlas. ROIs are annotated using numbers provided in Table 2. *A & B*. Positive connections. *C & D*. Negative connections.

Figure 8 shows the 171 positive (Figure 8 A & B) and 158 negative (Figure 8 C & D) significant connections observed in the rat data where no correction for global signal was used (as it was not needed since the distribution of the correlation values was centered approximately at zero). Each connection is shown as a colored line connecting two ROIs on coronal and axial rat-brain figures derived from the rat atlas [40] with ROIs numbers corresponding to the brain regions presented in Table 2.

Several common features across both species are evident from Figures 2 and 8. First, while many homologous bilateral regions (e.g. right and left anterior cingulate) have positive connections between each other, not even a single negative connection exists between homologous right and left hemispheric structures. Second, both negative and positive connections express a relatively high level of inter-hemispheric (non-homologous) symmetry. Note that in both the human and rat data, several regions had multiple positive connections, several had multiple negative connections and several had both multiple positive and negative connections (e.g., the right and left insula (#30, 31) in humans and the amygdala (#38 and 39) in rats), emphasizing the fact that Fisher-transformed values between regions reflect connection-specific rather than region-specific properties.

**Figure 9:**
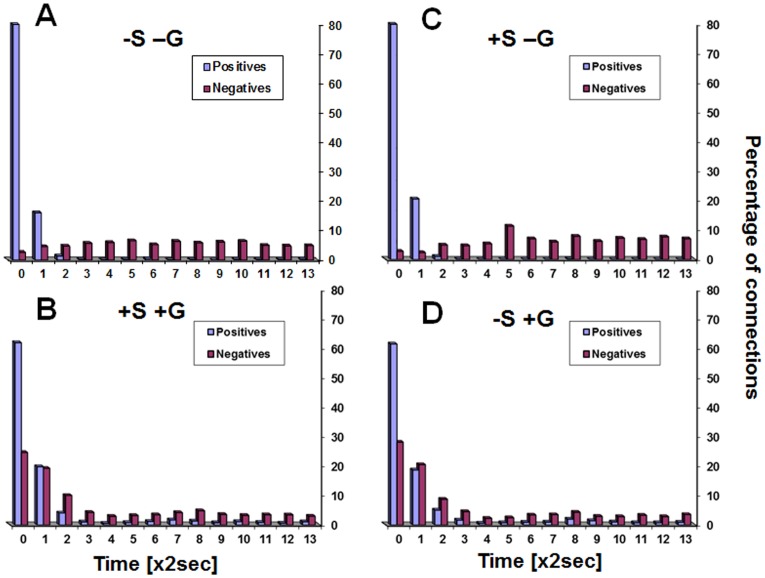
Effect of time-lags on correlation strengths in the human data. Percentage of connections that time-lags made more significant (more positive for positive connections and more negative for negative connections) at each time-lag value. *A*. Results for the analysis without smoothing and without global regression (‘–S -G’), *B*. Results for the analysis with smoothing and with global regression (‘+S +G’), *C*. Results for the analysis with smoothing and without global regression (‘+S -G’), *D*. Results for the analysis without smoothing and with global regression (‘–S +G’).

**Figure 10:**
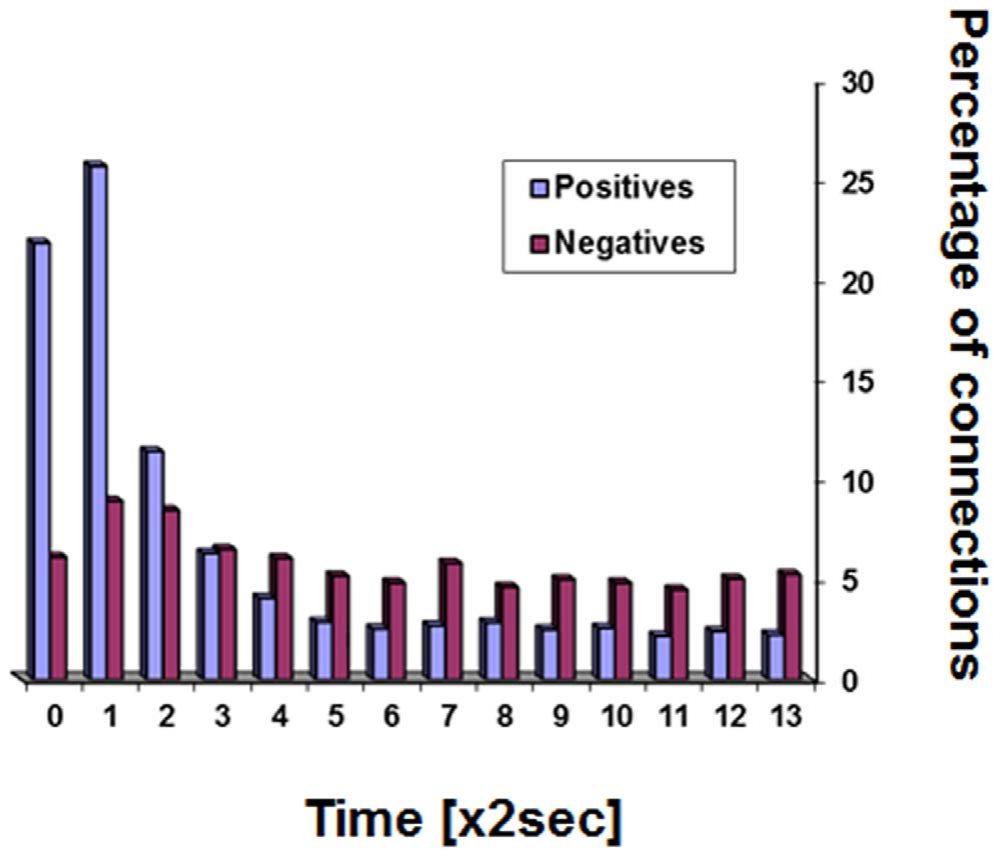
Effect of time-lags on correlation strengths in the rat data. Percentage of connections that time-lags made more significant (more positive for positive connections and more negative for negative connections) at each time-lag value.

Positive correlations between ROI pairs are obtained if ROI time courses express similar periodical behaviors without a time-lag. Negative correlations between ROI pairs can theoretically be obtained if both ROIs express similar periodical behaviors but with a time-lag between them. The effect of time–lags on the positive and the negative connections is presented in Figure 9 for humans and in Figure 10 for rats. The figures show the percentage of significant positive and negative connections that time-lags made them even more significant (higher Fisher-transformed values for positive connections and lower for negative connections). Since the calculations were performed on the data of each subject separately, a statistical comparison is possible. Table 4 gives the p-values for that comparison for each time-lag and each type of analysis. As seen in these Figures and in Table 4, time-lags of more than a few seconds reduced positive correlations while making negative correlations more negative. Specifically we note the following: (i) most positive connections were more significant with a zero time-lag, (ii) most negative connections were more significant with a non-zero time-lag, (iii) the time-lags that improve negative connections were of a wide range, (iv) the division between positive and negative connections was sharper when no correction for global signal was used, (v) the transition points from which any increase of the time-lag resulted in more negative than positive connections becoming more significant were at 4 sec in the human data and at 6 sec in the rat data, (vi) the use of global signal corrections increased the number of negative connections for which the most significant correlation was obtained using a zero time-lag (thus being similar to the positive connections). In contrast, the number of such ‘zero time-lag’ negative connections was negligible when no global signal correction was used. (vii) The rat data was qualitatively similar to human data.

**Table 4:** T-values for the comparison between the numbers of positive and negative connections that time-lag made more significance.

	-S-G	+S+G	+S-G	-S+G	rats
0	2.90369E-26	2.99046E-13	1.57702E-26	1.69953E-11	1.66596E-10
2	8.40016E-06	0.800385948	7.38712E-07	0.450100779	3.91086E-11
4	0.000407685	2.44899E-06	0.012682339	0.004888566	0.001371955
6	4.73828E-08	9.33376E-07	7.36576E-05	0.000790463	0.811502824
8	2.60049E-07	6.57005E-06	0.001934496	4.07728E-05	0.002860986
10	3.48352E-09	8.16819E-05	0.000272562	0.003190054	0.000538446
12	3.2188E-08	0.000238064	1.03808E-05	0.001122026	0.001629137
14	9.87039E-09	0.000522939	0.000942835	0.000260738	1.8236E-06
16	1.95238E-12	1.99754E-05	7.12566E-06	0.017933245	0.001564497
18	3.14229E-08	1.52653E-05	4.45951E-05	0.006811102	1.2003E-05
20	1.38689E-08	9.0614E-05	0.001227115	0.004817849	0.000457931
22	5.55054E-08	5.0776E-07	3.7641E-05	0.000158922	0.000431086
24	3.93325E-07	1.33919E-06	0.000267121	0.000903292	9.5524E-05
26	1.12124E-06	0.001065601	0.000725739	0.000401245	9.63E-06

More significance positive/negative connections were defined as connections with higher/lower Fisher transform values. Comparison was done for each time-lag and for the four human analyses as well as for the rat data. The rows are for different time-lags that are given is sec.

## Discussion

This study aimed to better understand the mechanisms underlying negative correlations in resting-state functional connectivity MRI. Analysis was performed on BOLD contrast temporal signals obtained from predefined anatomical ROIs. This type of analysis has several advantages, including a more robust signal-to-noise ratio (the average ROI signal was used), being less sensitive to registration and realignments errors, making the examination of symmetry between hemispheres possible and visualizing the functional connectivity organization on a broad and global level.

By comparing the positive and negative connections obtained through four different analysis methods applied on the human data, we demonstrated critical differences between positive and negative connections that were evident in all types of analysis. The major difference was that introducing non-zero time-lags between ROIs reduced the significance of the positive connections, while increasing the significance of the negative connections. In addition, positive and negative connections typically linked between different regional categories. For example, no negative connections were found linking two non-cortical structures. When comparing the different analyses, the following differences were observed: (i) spatial smoothing and correction for the global signal increased the number of significant positive connections while its effect on the negative connections was more complex (Figure 6), (ii) spatial smoothing and global signal correction had a small effect on positive connections with respect to their regional categorization and to the degree of their overlap, while the global signal correction had a major effect on negative connections, resulting in almost no overlap between connections that were significant with and without global signal correction (Figure 7 and Table 3), and (iii) the differential effect that time-lags had on positive and negative connections was the strongest when no global signal correction was applied.

When comparing the results from the human and the rat data, the patterns of inter-hemispheric symmetry and the prevalence of positive bilateral homologous connections were found to be similar. More importantly, negative connections in both species generally became more negative by introducing time-lags, while positive connections did not. It must however be emphasized that, in rodents, sedation was shown to yield superior *r-fcMRI* results compared to general anesthesia [44], suggesting that our rat data should be considered with caution. Additionally, no attempt was done to regress for cardiac and respiration pulsation although such filtering might have improved the results. We avoided doing this in order for the human and rat data processing to be as similar as possible. Nevertheless, we recently have shown significant *r-fcMRI* results using isoflurane anesthesia [45]–[47] and without cardiac and respiration regression, which strengthens our confidence in our rat data.

All these results support the assumption that the different forms of *r-*fcMRI correlations reflect different underlying physiological mechanism. We suggest a hypothesis that integrates the above findings into an inclusive model for understanding the mechanism of negative and positive connections. We are aware however, that the current evidence supporting this hypothesis is only circumstantial. The BOLD signal is affected by changes in both rCBF and rCBV. The coupling between CBF, CBV and the BOLD signal is complex, nonlinear, spatially inhomogeneous and even layer dependent [48]. The positive phase of the hemodynamic response function in response to stimulus is assumed to be dominated by rCBF changes, while the post stimulus undershoot is assumed to be affected by a *delayed* rCBV response [43], [49], [50]. An increase in rCBF presumably results in a decrease in blood deoxy-hemoglobin levels, causing an increase in the BOLD signal. In contrast, an increase in rCBV causes a total increase in deoxy-hemoglobin, resulting in a decrease of the BOLD signal. The balance between rCBF and rCBV could therefore determine the overall resulting BOLD signal (i.e., if the signal at any specific time point is above or below the baseline). We hypothesis that if the activity of two groups of neurons in two separate regions is highly synchronized, and if the hemodynamic response of one is rCBF dominated while that of the other is rCBV dominated, the temporal correlation between the BOLD signal in these regions will be negative. Moreover, since rCBV increases are delayed compared to rCBF increases, there will be a time-lag until the negative correlation reaches its maximal value. This view is based on the assumption that changes in BOLD signals during rest and following stimuli are comparable in magnitude [51] and mechanism. The long time-lags observed in the data (Figure 7) are in line with the reported post-stimulus length in humans [42] and in rats [43]. We further hypothesis that if the rCBV and/or the rCBF responses are spatially dependent (for example, synchronized neuronal activity resulting in an increase of rCBV in one region and a decrease in rCBV in another) one can also expect to find negative correlations with no time lags between such regions. Overall, we suggest that negative connections are the results of complex spatially inhomogeneous hemodynamic responses that are mediated by neuronal activity.

Our hypothesis is supported by several published findings suggesting that monoamines, mainly dopamine and serotonin, differentially affect CBV and CBF in different brain regions [52]–[57]. For example, it was shown that administration of agonists for the excitatory D_1_-like dopamine receptors causes an increase in CBV in certain regions, while agonists for the inhibitory D_2_-like receptors cause a decrease in CBV in other regions [57]. Such a relationship fits in well with the proposed CBV-based mechanism of negative connections, in which a positive synchronization between the activities of neurons within two regions that are differentially influenced by CBV will result in a negative correlation between their BOLD signals. Similarly, Shin et al. [56] demonstrated that in response to noxious electrical stimulation of the rat forepaw (known to induce endogenous dopaminergic neurotransmission), CBV was increased in the sensory cortex and at the same time was decreased in the caudate-putamen (CPu), although immunohistochemistry and electrophysiological recording demonstrated increased neuronal activity in the CPu.

The following findings lend further support to the hypothesis that differential neurovascular mechanisms are responsible for the positive and negative correlations between brain regions observed in r-fcMRI connectivity measurements: (i) All homologous bilateral connections were positive. Hemodynamic responses in homologous bilateral regions are expected to be similar (similar weightings of CBF and CBV). Consequently, the correlation between their BOLD signals is expected to be positive. (ii) Most of the negative connections became more significant when adding time-lags of a few seconds. Since rCBV changes were shown to be delayed compared to rCBF changes [43], [49], [50], BOLD signals that are affected by CBV are expected to be more time-delayed compared to BOLD signals affected by CBF. The observed range of ‘significance-optimizing’ time-lags matches the post-stimulus delays found in humans and rats. (iii) Similar findings were obtained for both human and rat data. Human and rat data were acquired by two different protocols, with different field strengths, at different arousal states, with different resolutions and were analyzed by different software algorithms. Their anatomy, hemodynamic responses and brain organization are different. In spite of all these, remarkable similarities between human and rat results were observed.

We recall that the definition of negative correlations is critical and the use of different definitions makes the comparison between studies difficult. For example, in a recent publication [58] mice *r-fcMRI* of BOLD and CBV weighed data, were compared. Similar independent component analysis (ICA) anti-correlated networks were observed with BOLD and with CBV data, opposing our hypothesis. However, it is likely that not finding significant negative connections in their ROI-based analysis results from the different definition of negative connection. Here we used a t-statistic distribution centered at zero to define positive and negative connections. As described above, the significant differences observed between positive and negative connections, as per this definition, strengthens our belief in its relevance.

In conclusion, we propose that positive and negative connections in *r-fcMRI* result from neuronal-mediated hemodynamic mechanisms leading to temporal and spatial heterogeneity in *r-fcMRI* BOLD responses. We suggest that positive connections are expected to be found between regions with synchronized neuronal activity and homogeneous hemodynamic responses while negative connections are expected to be found between regions with synchronized neuronal activity, yet with heterogeneous hemodynamic responses.

## References

[1] Buckner RL, Andrews-Hanna JR, Schacter DL (2008) The brain's default network: anatomy, function, and relevance to disease. Ann N Y Acad Sci 1124: 1–38. 18400922 10.1196/annals.1440.011

[2] Fox MD, Raichle ME (2007) Spontaneous fluctuations in brain activity observed with functional magnetic resonance imaging. Nat Rev Neurosci 8: 700–711. 17704812 10.1038/nrn2201

[3] Biswal B, Yetkin FZ, Haughton VM, Hyde JS (1995) Functional connectivity in the motor cortex of resting human brain using echo-planar MRI. Magn Reson Med 34(4): 537–541. 8524021 10.1002/mrm.1910340409

[4] Fox MD, Snyder AZ, Vincent JL, Corbetta M, Van Essen DC, et al (2005) The human brain is intrinsically organized into dynamic, anticorrelated functional networks. Proc Natl Acad Sci U S A 102: 9673–9678. 15976020 10.1073/pnas.0504136102 PMC1157105

[5] Greicius MD, Krasnow B, Reiss AL, Menon V (2003) Functional connectivity in the resting brain: a network analysis of the default mode hypothesis. Proc Natl Acad Sci U S A 100: 253–258. 12506194 10.1073/pnas.0135058100 PMC140943

[6] Raichle ME, MacLeod AM, Snyder AZ, Powers WJ, Gusnard DA, et al (2001) A default mode of brain function. Proc Natl Acad Sci U S A 98: 676–682. 11209064 10.1073/pnas.98.2.676 PMC14647

[7] Deco G, Jirsa VK, McIntosh AR (2011) Emerging concepts for the dynamical organization of resting-state activity in the brain. Nat Rev Neurosci 12: 43–56. 21170073 10.1038/nrn2961

[8] Damoiseaux JS, Rombouts SA, Barkhof F, Scheltens P, Stam CJ, et al (2006) Consistent resting-state networks across healthy subjects. Proc Natl Acad Sci U S A 103: 13848–13853. 16945915 10.1073/pnas.0601417103 PMC1564249

[9] Shehzad Z, Kelly AM, Reiss PT, Gee DG, Gotimer K, et al (2009) The resting brain: unconstrained yet reliable. Cereb Cortex 19: 2209–2229. 19221144 10.1093/cercor/bhn256 PMC3896030

[10] Zuo XN, Kelly C, Adelstein JS, Klein DF, Castellanos FX, et al (2010) Reliable intrinsic connectivity networks: test-retest evaluation using ICA and dual regression approach. Neuroimage 49: 2163–2177. 19896537 10.1016/j.neuroimage.2009.10.080 PMC2877508

[11] Wang K, van Meer MP, van der Marel K, van der Toorn A, Xu L, et al (2011) Temporal scaling properties and spatial synchronization of spontaneous blood oxygenation level-dependent (BOLD) signal fluctuations in rat sensorimotor network at different levels of isoflurane anesthesia. NMR Biomed 24: 61–67. 20669170 10.1002/nbm.1556

[12] Liu X, Zhu XH, Zhang Y, Chen W (2011) Neural origin of spontaneous hemodynamic fluctuations in rats under burst-suppression anesthesia condition. Cereb Cortex 21: 374–384. 20530220 10.1093/cercor/bhq105 PMC3020581

[13] Whitfield-Gabrieli S, Thermenos HW, Milanovic S, Tsuang MT, Faraone SV, et al (2009) Hyperactivity and hyperconnectivity of the default network in schizophrenia and in first-degree relatives of persons with schizophrenia. Proc Natl Acad Sci U S A 106: 1279–1284. 19164577 10.1073/pnas.0809141106 PMC2633557

[14] Castellanos FX, Margulies DS, Kelly C, Uddin LQ, Ghaffari M, et al (2008) Cingulate-precuneus interactions: a new locus of dysfunction in adult attention-deficit/hyperactivity disorder. Biol Psychiatry 63: 332–337. 17888409 10.1016/j.biopsych.2007.06.025 PMC2745053

[15] Chai XJ, Whitfield-Gabrieli S, Shinn AK, Gabrieli JD, Nieto Castanon A, et al (2011) Abnormal medial prefrontal cortex resting-state connectivity in bipolar disorder and schizophrenia. Neuropsychopharmacology 36: 2009–2017. 21654735 10.1038/npp.2011.88 PMC3158318

[16] Wang K, Liang M, Wang L, Tian L, Zhang X, et al (2007) Altered functional connectivity in early Alzheimer's disease: a resting-state fMRI study. Hum Brain Mapp 28: 967–978. 17133390 10.1002/hbm.20324 PMC6871392

[17] Grimm S, Boesiger P, Beck J, Schuepbach D, Bermpohl F, et al (2009) Altered negative BOLD responses in the default-mode network during emotion processing in depressed subjects. Neuropsychopharmacology 34: 932–943. 18536699 10.1038/npp.2008.81

[18] Kastrup A, Baudewig J, Schnaudigel S, Huonker R, Becker L, et al (2008) Behavioral correlates of negative BOLD signal changes in the primary somatosensory cortex. Neuroimage 41: 1364–1371. 18495495 10.1016/j.neuroimage.2008.03.049

[19] Kobayashi E, Bagshaw AP, Grova C, Dubeau F, Gotman J (2006) Negative BOLD responses to epileptic spikes. Hum Brain Mapp 27: 488–497. 16180210 10.1002/hbm.20193 PMC6871405

[20] Nakata H, Sakamoto K, Ferretti A, Gianni Perrucci M, Del Gratta C, et al (2009) Negative BOLD effect on somato-motor inhibitory processing: an fMRI study. Neurosci Lett 462: 101–104. 19576957 10.1016/j.neulet.2009.06.088

[21] Northoff G, Walter M, Schulte RF, Beck J, Dydak U, et al (2007) GABA concentrations in the human anterior cingulate cortex predict negative BOLD responses in fMRI. Nat Neurosci 10: 1515–1517. 17982452 10.1038/nn2001

[22] Pasley BN, Inglis BA, Freeman RD (2007) Analysis of oxygen metabolism implies a neural origin for the negative BOLD response in human visual cortex. Neuroimage 36: 269–276. 17113313 10.1016/j.neuroimage.2006.09.015 PMC2001204

[23] Schridde U, Khubchandani M, Motelow JE, Sanganahalli BG, Hyder F, et al (2008) Negative BOLD with large increases in neuronal activity. Cereb Cortex 18: 1814–1827. 18063563 10.1093/cercor/bhm208 PMC2790390

[24] Shmuel A, Yacoub E, Pfeuffer J, Van de Moortele PF, Adriany G, et al (2002) Sustained negative BOLD, blood flow and oxygen consumption response and its coupling to the positive response in the human brain. Neuron 36: 1195–1210. 12495632 10.1016/s0896-6273(02)01061-9

[25] Smith AT, Williams AL, Singh KD (2004) Negative BOLD in the visual cortex: evidence against blood stealing. Hum Brain Mapp 21: 213–220. 15038003 10.1002/hbm.20017 PMC6871689

[26] Shmuel A, Augath M, Oeltermann A, Logothetis NK (2006) Negative functional MRI response correlates with decreases in neuronal activity in monkey visual area V1. Nat Neurosci 9: 569–577. 16547508 10.1038/nn1675

[27] Fox MD, Zhang D, Snyder AZ, Raichle ME (2009) The global signal and observed anticorrelated resting state brain networks. J Neurophysiol 101: 3270–3283. 19339462 10.1152/jn.90777.2008 PMC2694109

[28] Murphy K, Birn RM, Handwerker DA, Jones TB, Bandettini PA (2009) The impact of global signal regression on resting state correlations: are anti-correlated networks introduced? Neuroimage 44: 893–905. 18976716 10.1016/j.neuroimage.2008.09.036 PMC2750906

[29] Weissenbacher A, Kasess C, Gerstl F, Lanzenberger R, Moser E, et al (2009) Correlations and anticorrelations in resting-state functional connectivity MRI: a quantitative comparison of preprocessing strategies. Neuroimage 47: 1408–1416. 19442749 10.1016/j.neuroimage.2009.05.005

[30] Chai XJ, Castanon AN, Ongur D, Whitfield-Gabrieli S (2011) Anticorrelations in resting state networks without global signal regression. Neuroimage 59: 1420–1428. 21889994 10.1016/j.neuroimage.2011.08.048 PMC3230748

[31] Bianciardi M, Fukunaga M, van Gelderen P, de Zwart JA, Duyn JH (2011) Negative BOLD-fMRI signals in large cerebral veins. J Cereb Blood Flow Metab 31: 401–412. 20859295 10.1038/jcbfm.2010.164 PMC3049531

[32] Chang C, Glover GH (2010) Time-frequency dynamics of resting-state brain connectivity measured with fMRI. Neuroimage 50: 81–98. 20006716 10.1016/j.neuroimage.2009.12.011 PMC2827259

[33] Carbonell F, Bellec P, Shmuel A (2011) Global and system-specific resting-state FMRI fluctuations are uncorrelated: principal component analysis reveals anti-correlated networks. Brain Connect 1: 496–510. 22444074 10.1089/brain.2011.0065 PMC3604782

[34] Devor A, Tian P, Nishimura N, Teng IC, Hillman EM, et al (2007) Suppressed neuronal activity and concurrent arteriolar vasoconstriction may explain negative blood oxygenation level-dependent signal. J Neurosci 27: 4452–4459. 17442830 10.1523/JNEUROSCI.0134-07.2007 PMC2680207

[35] Chao-Gan Y, Yu-Feng Z (2010) DPARSF: A MATLAB Toolbox for "Pipeline" Data Analysis of Resting-State fMRI. Front Syst Neurosci 4: 13. 20577591 10.3389/fnsys.2010.00013 PMC2889691

[36] Song XW, Dong ZY, Long XY, Li SF, Zuo XN, et al (2011) REST: a toolkit for resting-state functional magnetic resonance imaging data processing. PLoS One 6: e25031. 21949842 10.1371/journal.pone.0025031 PMC3176805

[37] Power JD, Barnes KA, Snyder AZ, Schlaggar BL, Petersen SE (2012) Spurious but systematic correlations in functional connectivity MRI networks arise from subject motion. Neuroimage 59: 2142–2154. 22019881 10.1016/j.neuroimage.2011.10.018 PMC3254728

[38] Maldjian JA, Laurienti PJ, Kraft RA, Burdette JH (2003) An automated method for neuroanatomic and cytoarchitectonic atlas-based interrogation of fMRI data sets. Neuroimage 19: 1233–1239. 12880848 10.1016/s1053-8119(03)00169-1

[39] Maldjian JA, Laurienti PJ, Burdette JH (2004) Precentral gyrus discrepancy in electronic versions of the Talairach atlas. Neuroimage 21: 450–455. 14741682 10.1016/j.neuroimage.2003.09.032

[40] Paxinos G, Watson C (2007) The Rat Brain in Stereotactic Coordinates 6th edition: Academic Press.

[41] Lowe MJ, mock BJ, Sorenson JA (1998) Functonal Connectivity in Single and multislice Echoplanar Imaging Using Resting-State Fluctuations. Neuroimage 7: 119–132. 9558644 10.1006/nimg.1997.0315

[42] Arichi T, Fagiolo G, Varela M, Melendez-Calderon A, Allievi A, et al (2012) Development of BOLD signal hemodynamic responses in the human brain. Neuroimage 63: 663–673. 22776460 10.1016/j.neuroimage.2012.06.054 PMC3459097

[43] Zong X, Kim T, Kim SG (2012) Contributions of dynamic venous blood volume versus oxygenation level changes to BOLD fMRI. Neuroimage 60: 2238–2246. 22401759 10.1016/j.neuroimage.2012.02.052 PMC3339492

[44] Kalthoff D, Po C, Wiedermann D, Hoehn M (2013) Reliability and spatial specificity of rat brain sensorimotor functional connectivity networks are superior under sedation compared with general anesthesia. NMR Biomed 26: 638–650. 23303725 10.1002/nbm.2908

[45] Lotan A, Lifschytz T, Lory O, Goelman G, Lerer B (2014) Amygdalar disconnectivity could underlie stress resilience in the Ahi1 knockout mouse: conclusions from a resting-state functional MRI study. Mol Psychiatry 19: 144. 24457523 10.1038/mp.2013.191

[46] Lotan A, Lifschytz T, Slonimsky A, Broner EC, Greenbaum L, et al (2014) Neural mechanisms underlying stress resilience in Ahi1 knockout mice: relevance to neuropsychiatric disorders. Mol Psychiatry 19: 243–252. 24042478 10.1038/mp.2013.123

[47] Goelman G, Ilinca R, Zohar I, Weinstock M (2014) Functional connectivity in prenatally stressed rats with and without maternal treatment with ladostigil, a brain-selective monoamine oxidase inhibitor. Eur J Neurosci. 10.1111/ejn.12621 24862938

[48] Goense J, Merkle H, Logothetis NK (2012) High-resolution fMRI reveals laminar differences in neurovascular coupling between positive and negative BOLD responses. Neuron 76: 629–639. 23141073 10.1016/j.neuron.2012.09.019 PMC5234326

[49] Buxton RB, Wong EC, Frank LR (1998) Dynamics of blood flow and oxygenation changes during brain activation: the balloon model. Magn Reson Med 39: 855–864. 9621908 10.1002/mrm.1910390602

[50] Buxton RB, Uludag K, Dubowitz DJ, Liu TT (2004) Modeling the hemodynamic response to brain activation. Neuroimage 23 Suppl 1 S220–233. 15501093 10.1016/j.neuroimage.2004.07.013

[51] Kenet T, Bibitchkov D, Tsodyks M, Grinvald A, Arieli A (2003) Spontaneously emerging cortical representations of visual attributes. Nature 425: 954–956. 14586468 10.1038/nature02078

[52] Chen YI, Choi JK, Xu H, Ren J, Andersen SL, et al (2010) Pharmacologic neuroimaging of the ontogeny of dopamine receptor function. Dev Neurosci 32: 125–138. 20523024 10.1159/000286215 PMC2919511

[53] Choi JK, Mandeville JB, Chen YI, Grundt P, Sarkar SK, et al (2010) Imaging brain regional and cortical laminar effects of selective D3 agonists and antagonists. Psychopharmacology (Berl) 212: 59–72. 20628733 10.1007/s00213-010-1924-6 PMC3822611

[54] Mueggler T, Razoux F, Russig H, Buehler A, Franklin TB, et al (2011) Mapping of CBV changes in 5-HT(1A) terminal fields by functional MRI in the mouse brain. Eur Neuropsychopharmacol 21: 344–353. 20656461 10.1016/j.euroneuro.2010.06.010

[55] Easton N, Marshall FH, Marsden CA, Fone KC (2009) Mapping the central effects of methylphenidate in the rat using pharmacological MRI BOLD contrast. Neuropharmacology 57: 653–664. 19733553 10.1016/j.neuropharm.2009.08.018

[56] Shih YY, Chen CC, Shyu BC, Lin ZJ, Chiang YC, et al (2009) A new scenario for negative functional magnetic resonance imaging signals: endogenous neurotransmission. J Neurosci 29: 3036–3044. 19279240 10.1523/JNEUROSCI.3447-08.2009 PMC6666445

[57] Choi JK, Chen YI, Hamel E, Jenkins BG (2006) Brain hemodynamic changes mediated by dopamine receptors: Role of the cerebral microvasculature in dopamine-mediated neurovascular coupling. Neuroimage 30: 700–712. 16459104 10.1016/j.neuroimage.2005.10.029

[58] Sforazzini F, Schwarz AJ, Galbusera A, Bifone A, Gozzi A (2014) Distributed BOLD and CBV-weighted resting-state networks in the mouse brain. Neuroimage 87: 403–415. 24080504 10.1016/j.neuroimage.2013.09.050

